# The EnergyPoplar project

**DOI:** 10.1186/1753-6561-5-S7-P113

**Published:** 2011-09-13

**Authors:** Magnus Hertzberg

**Affiliations:** 1 SweTree Technologies AB, Umeå, 904 03, Sweden

## 

Energy Poplar (Enhancing Poplar Traits for Energy Applications) is an EC Seventh Framework Programme project aimed at further improving poplar trees as an energy crop. The work is directed to understand and improve traits such as yield and wood properties coupled to Bioethanol production. The project also addresses environmental and economical sustainability questions.

The final goal of ENERGYPOPLAR is to develop poplar as a bioenergy short rotation coppice crop, suitable for large-scale deployment in Europe in areas unlikely to be used for food agricultural production. All will be placed in an environmental framework to ensure environmental sustainability with respect to land use, inputs and soil status

World primary energy consumption increased by 2.4% in 2007. With the worlds growing energy consumption, the development and use of renewable, sustainable liquid bio fuels has become a strategic priority for the EU. Bio fuels can minimize energy import dependence, reduce greenhouse gas emissions and assist rural and agricultural development.

Bio ethanol can be produced from energy crops that do not compete with food crops for land use and can be directly used by current transportation vehicles. This alcohol can be produced from biomass feedstock and in particular from cellulose, a sugar based polymer present in the cell wall of plants. Such crops are known as ‘second generation’ bio fuel crops.

To develop a new bio ethanol industry competitive with fossil fuels, the quality of the biomass feedstock, the methods to produce ethanol from cellulose and the yield per hectare must be improved. Energy trees must also support an environmentally sustainable agriculture that uses less agrochemicals, develops rural economies and spares natural forests from agricultural expansion.

Poplar is an economically and ecologically attractive energy crop because it displays a wide range of growth habits and can grow on marginal lands unsuited for food crops with reduced input costs and optimized land management. Additionally, poplar is a commercial crop and the model organism for hardwood tree genomics and physiology.

http://WWW.Energypoplar.eu for more information.

